# High incidence of medication documentation errors in a Swiss university hospital due to the handwritten prescription process

**DOI:** 10.1186/1472-6963-11-199

**Published:** 2011-08-18

**Authors:** Maximilian J Hartel, Lukas P Staub, Christoph Röder, Stefan Eggli

**Affiliations:** 1 Research Group for e-medication, Bern University Hospital, Inselspital, CH-3010 Berne, Switzerland; 2 Maurice E. Müller Research Centre, Institute for Evaluative Research, University of Berne, Stauffacherstrasse 78, CH-3014 Berne, Switzerland

**Keywords:** Medication safety, medication error, medical error, handwriting, legibility, adverse event

## Abstract

**Background:**

Medication errors have been reported to be a leading cause of death in hospitalized patients. In this study we focused on identifying and quantifying errors in the handwritten drug ordering and dispensing documentation processes which could possibly lead to adverse drug events.

**Methods:**

We studied 1,934 ordered agents (165 consecutive patients) retrospectively for medication documentation errors. Errors were categorized into: Prescribing errors, transcription errors and administration documentation errors on the nurses' medication lists. The legibility of prescriptions was analyzed to explore its possible influence on the error rate in the documentation process.

**Results:**

Documentation errors occurred in 65 of 1,934 prescribed agents (3.5%). The incidence of patient charts showing at least one error was 43%. Prescribing errors were found 39 times (37%), transcription errors 56 times (53%), and administration documentation errors 10 times (10%). The handwriting readability was rated as good in 2%, moderate in 42%, bad in 52%, and unreadable in 4%.

**Conclusions:**

This study revealed a high incidence of documentation errors in the traditional handwritten prescription process. Most errors occurred when prescriptions were transcribed into the patients' chart. The readability of the handwritten prescriptions was generally bad. Replacing the traditional handwritten documentation process with information technology could potentially improve the safety in the medication process.

## Background

In its 1999 landmark paper, "To Err is Human," the U.S. Institute of Medicine stressed the fact that medication errors are the eighth most frequent cause of death in the United States, more frequent than car accidents, breast cancer, or AIDS [1]. Adverse drug events, defined as injury caused by medical management involving drugs, are the most prevalent cause of inpatient injury and are often found to be preventable [2-5].

Dormann et al. reported adverse drug events (ADEs) to be the reason for 6% of hospital admissions in internal medicine in Germany, causing additional health care expenditures of several hundred million Euros. Almost half of the ADEs observed in that study were considered preventable [6]. Buajourdet et al. revealed an incidence of 18% fatal adverse drug events among inpatients who died in the Department for Internal Medicine in Norway [7]. A comprehensive study in the United States showed that almost every fifth medication dose reaching the patient is incorrect [8].

The drug prescription and administration process in most hospitals worldwide is still based on handwritten medical chart entries [2,9,10]. Several steps in this complex and unchecked process can harbour a high number of relevant errors. These undetected medication errors in patients' drug documentation may be a significant source of ADEs [11,12].

This study was performed to identify and quantify possible sources of error in the handwritten prescription and medication process in a large Swiss university hospital. Medical records were analyzed for readability of handwritten medication entries and for errors in the medication documentation. Possible strategies to reduce the incidence of errors in this high-risk process are discussed [13].

## Methods

### Study environment and patient sample

The investigation was conducted at the Surgical Department of the Inselspital University Hospital of Bern, Switzerland. The study sample consisted of all consecutive inpatients discharged from one 20-bed unit of the orthopedic department from January 1^st ^through January 31^st ^2005. The same unit was later used to carry out a software pilot with a newly developed computerized physician order entry system with clinical decision support. Ward staff consisted of up to 17 nurses. Sixteen residents were in charge of prescribing drugs to patients.

Although this observational study does not measure the effectiveness of an intervention to improve quality in health, we report the results, where appropriate, following the Standards for Quality Improvement Reporting Excellence (SQUIRE) [14,15]. This retrospective chart review complies with the regulations for retrospective research projects at the University Hospital Berne, agreed with the Cantonal Ethics Commission, Berne, Switzerland.

### The process of prescription and medication

**Figure 1:**
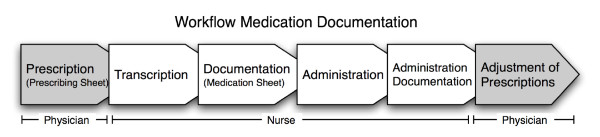
**Workflow of medication documentation in the Department for Orthopedic Surgery, Inselspital, Bern, Switzerland**.

**Figure 2:**
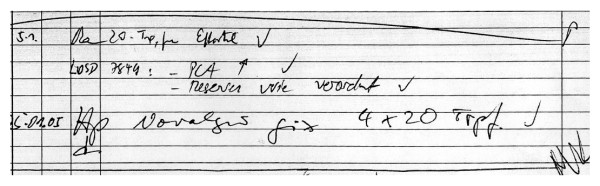
**Section of a prescribing sheet**. This shows the prescriptions of three different attending doctors.

Drug prescription and administration is a standardized process in our department and organized as follows (Figure 1): The treating physicians manually list all new prescriptions on a prescribing sheet, which is part of the patient chart and thus accessible to the responsible nurse (Figure 2).

**Figure 3:**
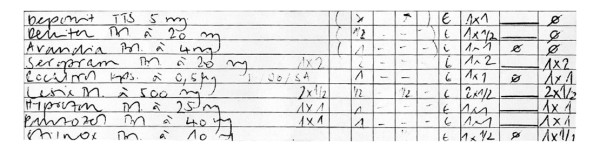
**Sample of a medication list**. Section of the oral medication that was paused for the day of the surgical intervention.

All prescriptions are then transcribed to standardized medication lists, on which the nursing staff documents the actual administration of drugs. Documented details include form, dose, route, frequency, and duration of drug administration. On a time scale, the applied dose and frequency for each day is expressed using figures and lines (Figure 3).

### Investigated Variables

#### Documentation Errors

Errors in medication documentation were categorized into three types:

1. Prescribing errors (found on prescribing sheets, e.g. wrong date, missing information about dosage),

2. Transcription errors (occur in the process of transcription from a prescribing sheet to a patient's medication list),

3. Administration documentation errors (errors in the documentation of the actual drug dispensation on the medication list by nursing staff).

#### Readability

Prescription sheets were analyzed for readability by three independent investigators: One senior surgeon, one resident, and a non-medical scientific associate. The readability of entries was categorized as good, moderate, bad, or unreadable. The inter-rater reliability of the initial ratings was calculated for the resulting classifications. Test-retest reliability was determined by 2 of the investigators by re-evaluating the readability of drug prescriptions on 30 randomly selected prescription sheets after at least three months. Cases of disagreement were resolved in a consensus meeting.

### Statistics

Due to the skewed distribution of data, we calculated Spearman's rank correlation coefficient ρ to estimate the association between the number of agents prescribed per patient, patient age and length of hospital stay.

Inter-rater reliability for the assessment of readability among the three raters was analyzed using Cohen's Kappa coefficient. Kappa is the proportion of agreements that is actually observed between raters, after adjusting for the proportion of agreements occurring by chance. A value of Kappa equal to +1 implies perfect agreement between the two raters, while that of -1 implies perfect disagreement. If Kappa has the value 0, there is no relationship between the ratings of the two raters, and any agreement or disagreement is due to chance alone. For the test-retest reliability between two raters, weighted Kappa coefficients were calculated. Agreement was qualified as follows: Low < 0.4, moderate 0.4-0.59, good 0.6-0.79, excellent > 0.79.

All statistical analyses were conducted using SAS 9.1 (SAS Institute, Inc., Cary, NC, USA).

## Results

### Sample characteristics

A total of 167 consecutive patient histories stored in the clinic information system in January 2005 were included in this study. In two cases the medication forms were missing. Thus, a total of 165 cases were considered valid for analysis. The median patient age was 55 years (range 17-93). Sixty-nine patients (42%) were female. The median length of stay was 6 days (range 1-53).

A total of 1,934 different agents were ordered in 576 different entries. The median number of agents per patient was 11 (range 1-30). Patient age and the number of prescribed drugs were positively correlated (ρ = 0.39; p < 0.0001). Furthermore, there was a positive correlation between the length of stay and the number of drugs prescribed (ρ = 0.67; p < 0.0001).

### Documentation Errors

A total of 105 errors were detected in 65 of the 1934 prescribed agents (3.5%). At least one error was found in the medication documentation of 71 of the 165 patients (43%), while in 94 cases no errors were found in the medication documentation (on both, the prescription sheets and medication lists). The median number of documentation errors per patient was 1 (range 0-9). The maximum number of 9 irregularities was detected in the drug documentation of an 86-year-old female patient who received 25 different agents during a 10-day stay. A detailed listing of the medication documentation errors found is depicted in Table 1.

**Table 1:** Listing of Medication Documentation Errors (n = 105), (Derived from previous work by Kenneth Barker et al 2002 [8])

Type of Error	Absolute Frequency	%
**Prescribing Errors**	**39**	**37.1**
Wrong/missing date/time	29	27.6
Missing dose specification	4	3.8
Ambiguous order/Overdose	3	2.9
Wrong patient	2	1.9
Missing frequency specification	1	1.0

**Transcription Errors**	**56**	**53.3**
Missing New Orders	28	26.7
Wrongly Transcribed Dose	18	17.1
Not Transcribed Stop Order	1	1.0
Others	9	8.6

**Administration documentation errors**	**10**	**9.5**
Omission or missing administration documentation	4	3.8
Wrong/missing date/time	2	1.9
Intentional alteration of original drug name	1	1.0
Inaccurate dosing documentation	1	1.0
Documentation on wrong patient sheet	1	1.0
Wrong categorization on sheet	1	1.0

**Total Errors**	**105**	**100.0**

Listing of Medication Documentation Errors (n = 105), (Derived from previous work by Kenneth Barker et al 2002 [8])

Prescribing errors occurred in 39 prescribed agents. Wrong or missing details about date and time were identified in 29 prescriptions (27.6%), which may have had a direct impact on the start time of a medication. In four cases (3.8%), the prescriptions were lacking dose information. Ambiguous orders, potentially leading to an overdose, were found three times (2.9%). One of these could potentially have resulted in an overdose with 18 mg of phenprocoumon (coumarine derivative) per day. In two cases (1.9%), the doctors wrote formally correct prescriptions but on the wrong patient's order sheet. Finally, the frequency of drug administration was missing in one prescription (1%).

Transcription errors occurred in 56 prescribed agents. Of these, 28 new drug orders (27%) were not transcribed to the medication list at all. In 18 cases (17%), the drug order was transcribed, but the dose was wrong in the medication list.

Administration documentation errors were found in 10 prescribed agents: In four cases (3.8%), the nursing staff failed to either document or entirely omitted administrations. In two cases (1.9%), the date and time were either missing or wrong. In one case (1%), the drug name was abbreviated and altered, in another a nurse was imprecise when documenting the dosing information. On one medication sheet the administration dispensing documentation of another patient was found. Finally, on one sheet the PRN nausea medication was categorized as PRN sleeping/agitation medication and vice versa.

### Readability

Figure 2 shows a section of a prescription sheet with prescriptions of three different doctors. Good readability was found in 2% of prescription sheets, moderate readability in 42%, and bad readability in 52%. Unreadable prescriptions were found in 4%. The agreement of the initial ratings between the three raters revealed the following pairwise Kappa values: 0.44 (95% CI 0.38-0.50), 0.33 (95% CI 0.27-0.39) and 0.36 (95% CI 0.30-0.41). Initial disagreement between the raters was only observed in prescriptions with moderate or bad readability, while the raters perfectly agreed upon unreadable prescriptions and those with good readability. The test-retest reliability for the two raters produced Kappa values of 0.65 (95% CI 0.53-0.77) and 0.60 (95% CI 0.49-0.71), respectively.

No documentation errors were found in prescription sheets with good readability. The median number of documentation errors in prescription sheets rated as moderately readable was 0.5 (range 0 - 4). In sheets with bad readability the median number of errors was 1 (range 0 -4).

## Discussion

This study revealed a high error rate in the prescription process in a large Swiss University Hospital. The process of transcribing a drug order manually from one sheet to another appears to be a significant source of error. More than half of the handwritten prescriptions were rated as poorly readable or unreadable.

It is well known that errors in the medication process can lead to adverse drug events [4,8,11,16,17]. Negligence and omission have been reported to be a prominent source of error, and transcription errors can potentially compromise patient outcomes [3,4,8,11,18]. In our study, at least one error in the prescription-transcription process was found in 3.5% of all prescribed agents. This was similar to the results published by Bobb in 2004 (6.2%) and Bates in 1995 (5.3%), whereas Barker found a markedly higher error rate of 19% [2,8,16]. Most errors found in our study were related to the transcription of prescriptions (53.3%). Others have reported lower transcription error rates (Bobb 2004: 4.9%; Leape 1995: 11.9%; Barker 2002, for omitted drug orders: 30.2%) [2,8,11]. These differences are mainly due to diverse definitions of error types and methodological approaches chosen by investigators based on the particular documentation practices in their health care system. Moreover, unlike other investigations, our study focussed on the handwritten medication documentation and its error proneness.

Compared to the data from Winslow et al. (20% illegible or readable with effort) and Aylamani et al., our study revealed an even worse legibility of the handwriting of physicians [19,20]. Agreement among the three raters in the readability evaluation was low, thus making evident that there are not only considerable differences in the quality of handwriting between different physicians but also between the subjective ability to read different handwritings. In addition, even when the two physicians rated the same prescriptions on two occasions, the agreement was not excellent. These findings are consistent with those from Alyamani et al., who also reported considerably low levels of illegibility (0.5%) in a consensus group of physicians. Interestingly, higher rates were found in the same study if the analysis was performed by nursing staff (2.5%) [20].

Different solutions to avoid errors in the handwritten prescription process are available. Omitting transcribing by having physicians write the prescriptions directly on medication lists removes one important source of errors. However, this does not solve the fundamental problem of poor readability of handwriting. Therefore, Computerized Physician Order Entry (CPOE) software solutions are increasingly taken into service and prove to eliminate multiple sources of error. In addition to the replacement of handwritten prescriptions by easily readable digital fonts, and the factual elimination of documentation errors, these software programs feature various security functions, such as checking for drug interactions, dosage levels, laboratory findings, and contraindications [2,13,21-23]. Of course, the use of CPOE systems cannot prevent every documentation error; it may even introduce new sources of error. For example, insufficient training of staff as well as poor quality of soft- and/or hardware have shown to facilitate additional error risks [10]. However, most of the data published supports the claim that CPOE systems provide a valuable solution for improving the quality of the medication process in hospitals [9,24-26].

This study has some limitations. It is a retrospective evaluation and, therefore, discussions between doctors and nurses concerning patient medication could not be measured. However, an unblinded prospective approach may lead to exceptionally careful staff behavior and over-accurate medication documentation as a result of being supervised. According to Alyamani et al., the level of illegibility identified by nurses may be higher compared to the analysis of a consensus group [20]. Hence, the level of illegibility found may have differed if a nurse had participated in the investigators group. This study was conducted on one ward in a single institution, limiting the generalizability of the findings presented in this paper. Further studies should involve non-surgical departments that treat patients with complex medical conditions requiring multiple drugs and long hospital stays.

## Conclusion

In conclusion, this study found a high error rate in the medication process in a university hospital. Replacing the traditional handwritten documentation process with information technology may potentially improve the quality of the medication process.

## Competing interests

None of the authors report having competing interests that could inappropriately influence the content of this work.

## Authors' contributions

MJH was involved in the conception of the paper, literature review, manuscript preparation, editing and submission. LPS performed the statistical analyses and contributed to the conception and editing of the paper. CR was involved in the manuscript preparation and editing of the paper. SE conceived the idea of the study. All authors contributed to the editing and review of the manuscript and gave their approval of the final manuscript.

## Pre-publication history

The pre-publication history for this paper can be accessed here:

http://www.biomedcentral.com/1472-6963/11/199/prepub

## References

[B1] Kohn LT Corrigan JM Donaldson MS To Err Is Human: Building a Safer Health System 1999 Washington, D.C.: National Academies Press 25077248

[B2] Bobb A Gleason K Husch M Feinglass J Yarnold PR Noskin GA The epidemiology of prescribing errors: the potential impact of computerized prescriber order entry Arch Intern Med 2004 164 7 785 792 10.1001/archinte.164.7.785 15078649

[B3] Brennan TA Leape LL Laird NM Hebert L Localio AR Lawthers AG Newhouse JP Weiler PC Hiatt HH Incidence of adverse events and negligence in hospitalized patients. Results of the Harvard Medical Practice Study I N Engl J Med 1991 324 6 370 376 10.1056/NEJM199102073240604 1987460

[B4] Leape LL Brennan TA Laird N Lawthers AG Localio AR Barnes BA Hebert L Newhouse JP Weiler PC Hiatt H The nature of adverse events in hospitalized patients. Results of the Harvard Medical Practice Study II N Engl J Med 1991 324 6 377 384 10.1056/NEJM199102073240605 1824793

[B5] Kopp BJ Erstad BL Allen ME Theodorou AA Priestley G Medication errors and adverse drug events in an intensive care unit: direct observation approach for detection Crit Care Med 2006 34 2 415 425 10.1097/01.CCM.0000198106.54306.D7 16424723

[B6] Dormann H Neubert A Criegee-Rieck M Egger T Radespiel-Troger M Azaz-Livshits T Levy M Brune K Hahn EG Readmissions and adverse drug reactions in internal medicine: the economic impact J Intern Med 2004 255 6 653 663 10.1111/j.1365-2796.2004.01326.x 15147529

[B7] Buajordet I Ebbesen J Erikssen J Brors O Hilberg T Fatal adverse drug events: the paradox of drug treatment J Intern Med 2001 250 4 327 341 10.1046/j.1365-2796.2001.00892.x 11576320

[B8] Barker KN Flynn EA Pepper GA Bates DW Mikeal RL Medication errors observed in 36 health care facilities Arch Intern Med 2002 162 16 1897 1903 10.1001/archinte.162.16.1897 12196090

[B9] Ash JS Gorman PN Seshadri V Hersh WR Computerized physician order entry in U.S. hospitals: results of a 2002 survey J Am Med Inform Assoc 2004 11 2 95 99 14633935 10.1197/jamia.M1427 PMC353025

[B10] Koppel R Metlay JP Cohen A Abaluck B Localio AR Kimmel SE Strom BL Role of computerized physician order entry systems in facilitating medication errors Jama 2005 293 10 1197 1203 10.1001/jama.293.10.1197 15755942

[B11] Leape LL Bates DW Cullen DJ Cooper J Demonaco HJ Gallivan T Hallisey R Ives J Laird N Laffel G Nemeskal R Petersen LA Porter K Servi D Shea B Small SD Sweitzer BJ Taylor Thompson B Vander Vliet M Group APS Leape L Servi D Laird N Bates DW Hojonski-Diaz P Petersen LA Petrycki S Vander Vliet M Cotugno M Patterson H Systems analysis of adverse drug events. ADE Prevention Study Group Jama 1995 274 1 35 43 10.1001/jama.274.1.35 7791256

[B12] Barber N Rawlins M Dean Franklin B Reducing prescribing error: competence, control, and culture Qual Saf Health Care 2003 12 Suppl 1 i29 32 14645746 10.1136/qhc.12.suppl_1.i29 PMC1765766

[B13] Grandt D Friebel H Müller-Oerlinghausen B Arzneitherapie(un)sicherheit: Notwendige Schritte zur Verbesserung der Patientensicherheit bei medikamentöser Therapie Deutsches Ärzteblatt 2005 102 821858484

[B14] Davidoff F Batalden P Stevens D Ogrinc G Mooney S Publication guidelines for quality improvement in health care: evolution of the SQUIRE project Qual Saf Health Care 2008 17 Suppl 1 i3 9 10.1136/qshc.2008.029066 18836063 PMC2773518

[B15] Ogrinc G Mooney SE Estrada C Foster T Goldmann D Hall LW Huizinga MM Liu SK Mills P Neily J Nelson W Pronovost PJ Provost L Rubenstein LV Speroff T Splaine M Thomson R Tomolo AM Watts B The SQUIRE (Standards for QUality Improvement Reporting Excellence) guidelines for quality improvement reporting: explanation and elaboration Qual Saf Health Care 2008 17 Suppl 1 i13 32 10.1136/qshc.2008.029058 18836062 PMC2602740

[B16] Bates DW Boyle DL Vander Vliet MB Schneider J Leape L Relationship between medication errors and adverse drug events J Gen Intern Med 1995 10 4 199 205 10.1007/BF02600255 7790981

[B17] Gurwitz JH Field TS Judge J Rochon P Harrold LR Cadoret C Lee M White K LaPrino J Erramuspe-Mainard J DeFlorio M Gavendo L Auger J Bates DW The incidence of adverse drug events in two large academic long-term care facilities Am J Med 2005 118 3 251 258 10.1016/j.amjmed.2004.09.018 15745723

[B18] Brennan TA Hebert LE Laird NM Lawthers A Thorpe KE Leape LL Localio AR Lipsitz SR Newhouse JP Weiler PC Hiatt H Hospital characteristics associated with adverse events and substandard care Jama 1991 265 24 3265 3269 10.1001/jama.265.24.3265 2046108

[B19] Winslow EH Nestor VA Davidoff SK Thompson PG Borum JC Legibility and completeness of physicians' handwritten medication orders Heart Lung 1997 26 2 158 164 10.1016/S0147-9563(97)90076-5 9090521

[B20] Alyamani NA Hopf Y Williams DJ Prescription quality in an acute medical ward Pharmacoepidemiol Drug Saf 2009 18 12 1158 1165 10.1002/pds.1830 19670357

[B21] Bates DW Leape LL Cullen DJ Laird N Petersen LA Teich JM Burdick E Hickey M Kleefield S Shea B Vander Vliet M Seger DL Effect of computerized physician order entry and a team intervention on prevention of serious medication errors Jama 1998 280 15 1311 1316 10.1001/jama.280.15.1311 9794308

[B22] Kuperman GJ Gibson RF Computer physician order entry: benefits, costs, and issues Ann Intern Med 2003 139 1 31 39 12834316 10.7326/0003-4819-139-1-200307010-00010

[B23] Bates DW Teich JM Lee J Seger D Kuperman GJ Ma'Luf N Boyle D Leape L The impact of computerized physician order entry on medication error prevention J Am Med Inform Assoc 1999 6 4 313 321 10.1136/jamia.1999.00660313 10428004 PMC61372

[B24] Burt CW Hing E Use of computerized clinical support systems in medical settings: United States, 2001-03 Adv Data 2005 353 1 8 15759905

[B25] Nightingale PG Adu D Richards NT Peters M Implementation of rules based computerised bedside prescribing and administration: intervention study Bmj 2000 320 7237 750 753 10.1136/bmj.320.7237.750 10720357 PMC27317

[B26] Upperman JS Staley P Friend K Neches W Kazimer D Benes J Wiener ES The impact of hospitalwide computerized physician order entry on medical errors in a pediatric hospital J Pediatr Surg 2005 40 1 57 59 10.1016/j.jpedsurg.2004.09.024 15868559

